# Necdin Protects Embryonic Motoneurons from Programmed Cell Death

**DOI:** 10.1371/journal.pone.0023764

**Published:** 2011-09-02

**Authors:** Julianne Aebischer, Rachel Sturny, David Andrieu, Anne Rieusset, Fabienne Schaller, Sandrine Geib, Cédric Raoul, Françoise Muscatelli

**Affiliations:** 1 Inserm-Avenir, Mediterranean Institute of Neurobiology, INMED, Marseille, France; 2 Université d'Aix-Marseille, Faculté des Sciences, Marseille, France; 3 Developmental Biology Institute of Marseille Luminy, IBDML, Marseille, France; 4 Inserm U901, Mediterranean Institute of Neurobiology, INMED, Campus scientifique de Luminy, Marseille, France; French National Centre for Scientific Research, France

## Abstract

*NECDIN* belongs to the type II Melanoma Associated Antigen Gene Expression gene family and is located in the Prader-Willi Syndrome (PWS) critical region. *Necdin*-deficient mice develop symptoms of PWS, including a sensory and motor deficit. However, the mechanisms underlying the motor deficit remain elusive. Here, we show that the genetic ablation of *Necdin*, whose expression is restricted to post-mitotic neurons in the spinal cord during development, leads to a loss of 31% of specified motoneurons. The increased neuronal loss occurs during the period of naturally-occurring cell death and is not confined to specific pools of motoneurons. To better understand the role of *Necdin* during the period of programmed cell death of motoneurons we used embryonic spinal cord explants and primary motoneuron cultures from *Necdin*-deficient mice. Interestingly, while *Necdin*-deficient motoneurons present the same survival response to neurotrophic factors, we demonstrate that deletion of *Necdin* leads to an increased susceptibility of motoneurons to neurotrophic factor deprivation. We show that by neutralizing TNFα this increased susceptibility of *Necdin*-deficient motoneurons to trophic factor deprivation can be reduced to the normal level. We propose that Necdin is implicated through the TNF-receptor 1 pathway in the developmental death of motoneurons.

## Introduction

The human *NECDIN* gene is one of the five genes that have been associated with the Prader-Willi syndrome (PWS) [1], [2], [3], a rare genetic neurodevelopmental disease characterized by a variety of physical, cognitive, and behavioral defects. The most significant characteristics at birth are feeding problems, severe hypotonia, breathing alterations and hypogonadism. Later, PW infants become obese, present a short stature, a motor delay and cognitive deficits [4]. A potential role of NECDIN in the etiology of PWS is supported by studies of three mouse models [5], [6], [7] in which the *Necdin* gene has been inactivated resulting in alterations of breathing and behavior, defects of the sensory system and in the hypothalamic nuclei. Similar alterations are described in Prader-Willi patients suggesting that NECDIN is responsible for specific Prader-Willi symptoms.

Necdin belongs to the type II Melanoma-Associated antigen Gene Expression (MAGE) family that shares a MAGE homology domain [8], which has recently been shown to bind RING proteins to form active E3 Ubiquitin Ligases [9]. Necdin locates in the nucleus and/or cytoplasm and has been reported to interact with cell cycle proteins (p53, E2F1, E2F2) [10], [11], [12], [13], [14], transmembrane proteins (p75^NTR^, TrkA, Nogo) [7], [15], [16], [17], and cytoplasmic interactors (MAGED1, FEZ1, BBS4, NEFA) [18], [19]. Through these interactions, Necdin has been proposed to participate in a broad range of biological activities including cell growth, migration, differentiation and cell death/survival (see for review [20]). However the precise molecular function as well as the physiological relevance of Necdin in those processes remains largely unknown.

In the mouse nervous system, *in vivo* studies demonstrated Necdin involvement in cellular migration, axonal outgrowth and fasciculation processes [19], [21], [22], [23], [24], [25] as well as in neuronal apoptosis [14], [23], [26], [27]. We and others documented an anti-apoptotic function of Necdin in developing sensory neurons of the dorsal root ganglia (DRG) [7], [26], [27]. Interestingly, we showed that its anti-apoptotic function is restricted to a subpopulation of sensory neurons. Indeed, we showed that the abrogation of Necdin, in the lumbar region, triggered a 40% increase of post-mitotic apoptosis during the embryonic wave of naturally occurring cell death. This additional cell death resulted in a 30% loss of specified TrkA (nociception) and TrkC (proprioception) sensory neurons although the TrkB population (mechanoreception) was not modified. Beside the sensory deficits previously described in mutant mice, our study suggested that motor functions might also be deficient [27].

Here, we have further investigated the contribution of Necdin in the development of spinal motoneurons. We found that the genetic deletion of *Necdin* leads to the loss of lumbar motoneurons. This loss is due to an increase death of motoneuron during the wave of naturally-occurring cell death, which is predominantly dependent on the accessibility to neurotrophic support. Interestingly, we show that *Necdin-*deficient motoneurons present an increased susceptibility to neurotrophic factor (NTF) deprivation, which involves the activation of the tumor necrosis factor alpha (TNFα)/TNF receptor (TNFR) signaling pathway. Overall, our findings strongly suggest a developmental cause of motor deficit observed in PW patients.

## Results

### Targeted deletion of *Necdin* in mice leads to hindlimb motor deficits

Because Necdin is an imprinted gene, only paternally expressed, we crossed heterozygote males (*Necdin* ^−m/+p^) with wildtype C57BL/6J females, in the generated litters, half the embryos were control (*Necdin* ^+/+^) and half were functionally *Necdin-*deficient (*Necdin^+m/−p^*).

Previous phenotypical studies revealed alterations of the sensory system in *Necdin*-deficient mice but also suggested a motor deficit [27]. Indeed, we observed that from birth, *Necdin* pups and young mutant (10-day-old) mice display obvious problems in moving and flexing their hindpaws (Figure S1), although this deficit cannot be quantified at this age. Although, this motor problem tends to diminish over time, we were able to confirm that adult *Necdin*-deficient mice have decreased motor performance in an accelerating rotarod test compared to age-matched wildtype mice (Figure S1 and Text S1, Supporting materials and methods). These results show that *Necdin*-deficient mice have a motor deficit starting at birth, suggesting a developmental problem.

### Necdin is expressed in post-mitotic motoneurons during development and at postnatal ages

**Figure 1:**
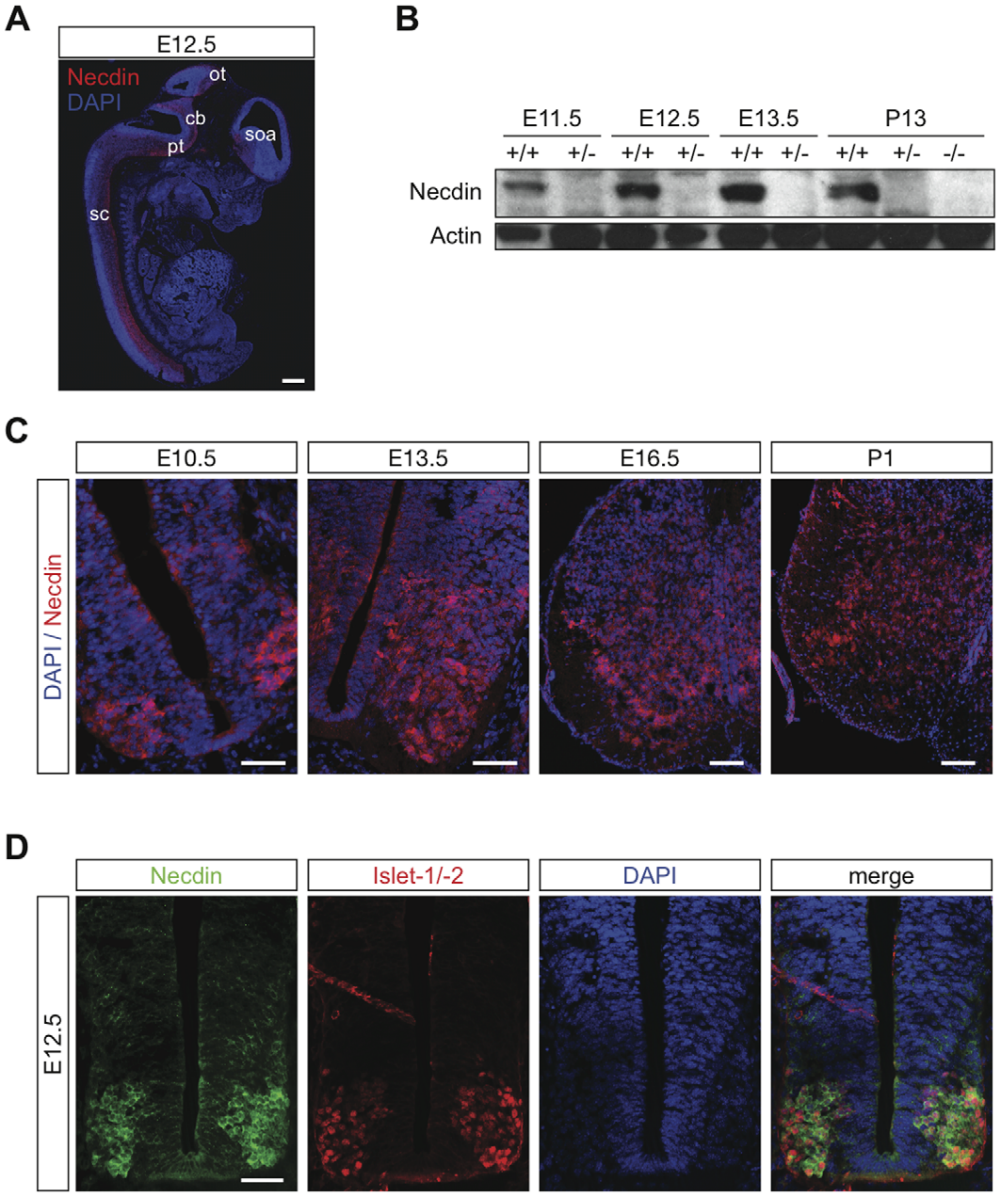
Necdin is expressed in motoneurons during embryonic development and postnatal stage. (A) Sagital section of whole E12.5 mouse embryo immunostained with a Necdin specific antibody (in red). Section is stained with DAPI (in blue). sc, spinal cord; pt, pyramidal tract; cb, cerebellum; ot, optic tract and soa, supraoptic area. (B) Western blot analysis of Necdin levels in the ventral horn of the lumbar spinal cord at indicated stages. Actin served as a loading control. In absence of the paternal copy of the Necdin gene (the maternal copy is silent due to imprinting mechanism) or in homozygous animals deleted for both copies, Necdin expression is not detected. P, Post-natal day. (C) Immunohistochemical analysis of Necdin expression (in red) in the spinal cord at different stages. Transverse sections were counter-stained with DAPI (in blue). (D) Necdin expression (in green) is mainly restricted to motoneurons, as identified by Islet-1/-2 immunostaining (in red). Later (C), its expression is extended to other cells (E.13.5, E16.5 and P1). Scale bar: A: 500 µm and C, D: 100 µm.

We previously described an expression of *NECDIN* gene in the large ventral horn neurons of the spinal cord from 10-week-old human embryo [2]. Then, in order to investigate the mechanisms underlying motor deficit observed in *Necdin* mutant mice, we first studied the expression pattern of Necdin in the spinal cord at different developmental and post-natal stages. Using Necdin specific antibodies, we observed Necdin expression along the rostro-caudal axis of the spinal cord at embryonic day 10.5 post-*coïtum* (E10.5), which becomes obvious at E12.5 (Figure 1A, S2A). Necdin expression in the spinal cord is maintained throughout embryonic development and is present after birth (Figure 1B,C). Necdin is distributed in a homogeneous way in all motoneuron columns. With regard to the Necdin phenotype, we focused our attention on the lumbar region and showed that at E10.5 and E12.5 Necdin is exclusively expressed in the ventral horn of the spinal cord (Figure 1C,S2A). Furthermore, at these stages, Necdin-immunoreactive cells were identified as motoneurons as demonstrated by co-immunolabeling with Islet-1/-2 (Figure 1D) and Hb9 (Figure S2A). These double labeling experiments also confirmed that all the developing post-mitotic motoneurons express Necdin. From E13.5, Necdin expression was increased in the medio-ventral part of the spinal cord, and at E16.5 and P1, we observed an extension of this pattern to the central and dorsal region (Figure 1C). We confirmed using in situ hybridization that the expression of *Necdin* mRNA was consistent with the developmental expression profile of the protein (Figure S2B,C). Thus, Necdin appeared to be expressed first in developing post-mitotic motoneurons and progressively in others cell types.

### Loss of lumbar specified motoneurons in *Necdin*-deficient mice during embryonic development

**Figure 2:**
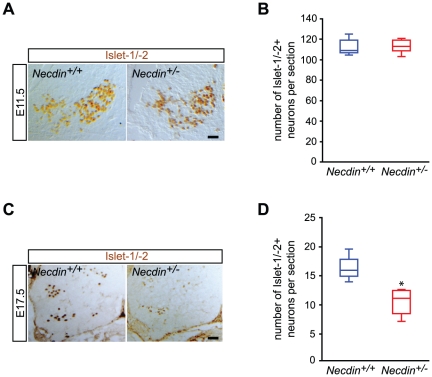
Loss of lumbar specified motoneurons during embryonic development. Transverse sections of E11.5 (A) and E17.5 (C) lumbar spinal cords were immunolabeled with anti-Islet-1/-2 antibodies. (A) and (B) At E11.5, when pools of post-mitotic motoneurons are generated, no significant difference in the number of Islet-1/-2 positive cells/section is detected between the ventral horn spinal cords of mutant (*Necdin^+/−^*) and control (*Necdin^+/+^*) mice. (C) and (D) At E17.5, when motoneurons are specified, a significant difference in the number of Islet-1/-2 positive cells/section is observed between both genotypes, with a loss of 31% of cells in *Necdin*-deficient mice (*Necdin^+/−^*). Scale bar, 50 µm.

We next looked for cellular defects in motoneuron development in *Necdin*-deficient mice. We studied two embryonic stages: E11.5, when the pool of developing postmitotic motoneurons is established [28], [29], [30] and E17.5 at the end of the period of programmed cell death (PCD), when all embryonic motoneurons are specified. At E11.5, we observed a similar number of Islet-1/-2 positive neurons between wildtype and mutant embryos (*Necdin^+/+^*: 109 (106, 119), *n* = 4; *Necdin* ^+/−^: 113 (108, 119), *n* = 4; non significant, n.s) (Figure 2A,B).

In contrast we performed a quantitative analysis of the Islet-1/-2 positive neurons in the ventral horns of E17.5 lumbar spinal cords, we found a significant reduction of 31% of Islet-1/-2 positive cells in *Necdin-*deficient compared to wildtype spinal cords (*Necdin^+/+^*: 16 (15, 18), *n* = 4; *Necdin* ^+/−^: 11 (8, 12), *n* = 4; *P*<0.05) (Figure 2C,D). We showed that this reduction of Islet-1/-2 positive neurons was specific to motoneurons since the number of the medio-ventral sub-population of Islet-1/-2 positive interneurons, did not differ between *Necdin*-deficient and wildtype mice at E17.5 (Figure S3) (*Necdin^+/+^*: 29 (29, 30), *n* = 4; *Necdin* ^+/−^: 28 (27, 30), *n* = 4; n.s).

The loss of motoneurons observed in the lumbar region of *Necdin*-deficient mice at E17.5, but not at E11.5, stages suggests that the genetic deletion of *Necdin* does not influence the generation of post-mitotic motoneurons, but rather might act during the period of naturally-occurring cell death that takes place between E12.5 and E15.5 in lumbar spinal cord.

### Necdin deficiency leads to an increase of lumbar motoneuron death during the period of programmed cell death

**Figure 3:**
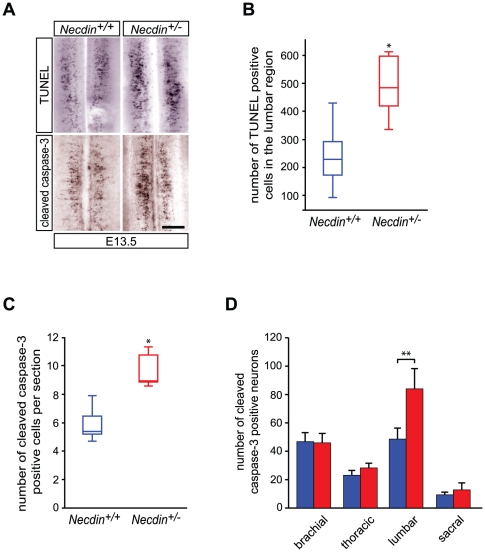
Increased apoptotic motoneuron cell death in the lumbar regions of mutant embryos compared to wildtypes. A wave of motoneuron developmental cell death is known to occur normally in the lumbar region between the embryonic stages E11.5 and E14.5. (A) Illustration of TUNEL and activated caspase-3 labeling on whole mount spinal cord, focused on the lumbar region at E13.5. (B) Quantification of TUNEL positive cells in the lumbar whole mount spinal cord reveals a significant increase of TUNEL positive cells in mutant (*Necdin^+/−^*)(*n* = 5) compared to wildtype (*Necdin^+/+^*) embryos (*n* = 5). (C) Quantification of activated caspase-3 positive cells on serial transverse sections of the lumbar spinal cord at E13.5 show a significant increase of activated-Casapse-3 positive cells/section in mutant (*Necdin^+/−^*) mice (*n* = 5) compared with the wildtype (*Necdin^+/+^*) embryos (*n* = 5). (D) Quantification of activated caspase-3 positive cells at different levels (brachial, thoracic, lumbar and sacral regions) of the spinal cord was done on whole mount spinal cord immunolabeled with anti-cleaved caspase-3 antibody (*n* = 3). Data in (B) and (C) are plotted in box-and-whisker format. Values in (D) represent means ± standard deviation (S.D). Scale bar, 100 µm, (*P<0.05 and **P<0.01).

We then asked whether Necdin plays a role in the survival of motoneurons during the period of PCD. Towards this goal, we first compared the number of apoptotic cells, between wildtype and *Necdin*-deficient embryonic spinal cords using a longitudinal whole-mount terminal deoxyribonucleotidyl transferase-mediated dUTP-digoxigenin nick end labeling analysis (TUNEL) [30]. At E11.5, very few apoptotic nuclei were detected in wildtype and mutant mice. At E12.5, apoptotic nuclei were present in controls and *Necdin* mutants (data not shown). At E13.5, when a peak of normal motoneuron PCD occurs [30], a significant increase of about 50% of the total number of TUNEL-positive cells was observed in *Necdin*-deficient spinal cord (*Necdin* ^+/+^: 229 (194, 251), *n* = 5; *Necdin* ^+/−^: 484 (442, 505), *n* = 5; *P*<0.05) (Figure 3 A,B). At E15.5, when PCD is nearly complete [30], TUNEL staining was reduced to few cells in the wildtype as well as in mutant spinal cords (data not shown). Using double staining, we then confirmed that, in both wildtype and mutant spinal cords, TUNEL-positive nuclei were Islet-1/-2 positive. Interestingly Islet-1/-2 staining revealed that the TUNEL signal was not restricted to specific pools of motoneurons in both *Necdin*-deficient and wildtype embryos (data not shown). To confirm that *Necdin* deficiency leads to increased neuronal death during the period of PCD, we quantified the number of cleaved caspase-3 positive cells, at E13.5, in *Necdin*-deficient and wildtype lumbar spinal cords. Consistently, we observed a significant 41% increase of cleaved caspase-3 positive cells in *Necdin*-mutant mice compared to wildtype mice (*Necdin* ^+/+^: 5.3 (5.1, 6.4), *n* = 5; *Necdin* ^+/−^: 8.9 (8.8, 10.7), *n* = 5; *P*<0.05) (Figure 3A,C). Therefore, the loss of lumbar motoneurons observed in *Necdin* mutants at E17.5 could be attributed to an increase in cell death during the period of developmental cell death. Notably, at E13.5, when the peak of PCD occurs in the brachial, thoracic, lumbar and sacral region [30], no significant difference was observed in the total number of cleaved caspase-3 positive cells in the brachial, thoracic or sacral regions of the spinal cord in wildtype embryos compared to mutant embryos (Figure 3D). In addition, we determined the number of lumbar motoneurons in 11-day-old *Necdin*-deficient and wildtype mice. We observed a significant difference of about 27% between *Necdin^+/+^* and *Necdin^+/−^* surviving motoneurons (*Necdin* ^+/+^: 1492 (1387, 1558), *n* = 3; *Necdin* ^+/−^: 1080 (1050, 1199), *n* = 3; *P*<0.05). This difference is similar to the one observed at E17.5. These results suggest that Necdin has a prosurvival function during early stages of lumbar motoneuron development.

### The increased motoneuron death in *Necdin*-deficient spinal cord explants does not depend on neurotrophic support

**Figure 4:**
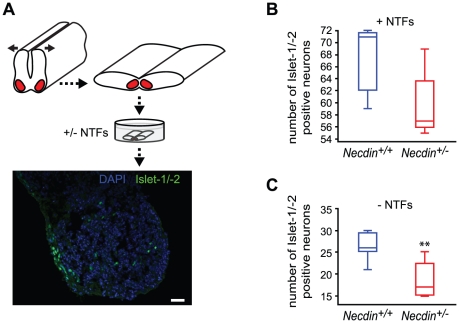
Role of trophic factors in motoneuron survival of *Necdin*-deficient E12.5 embryos. (A) Dissections of the lumbar spinal cord regions were performed at E12.5 and the explants were cultured in the presence or in the absence of NTFs for 2 days. Cryostat transverse sections of explants were then immunolabeled using Islet-1/-2 antibodies (green) in order to define the number of surviving motoneurons per explant. (B) In the presence of NTFs (+NTFs), the number of Islet-1/-2 positive cells is not significantly higher in mutant (*Necdin^+/−^*) explants (*n* = 5) compared with wildtype (*Necdin^+/+^*) explants (*n* = 5). (C) In the absence of NTFs (−NTFs), a significant 35% decrease of Islet-1/-2 positive cells is observed in *Necdin-*deficient (*Necdin^+/−^*) explants (*n* = 8) compared to wildtype (*Necdin^+/+^*) explants (*n* = 8), suggesting that the increase of cell death in *Necdin-*deficient motoneurons is not dependant on NTFs. Scale bar: 100 µm.

Our next goal was to investigate whether Necdin acts by “potentiating” the action of trophic factors (first hypothesis) or by interfering with extrinsic signals (second hypothesis), mediated by death receptors, which can be activated following NTF deprivation [31]. In the first hypothesis, we should observe, in the presence of trophic factors, an increased motoneuron death in *Necdin*-deficient explants compared to wildtype explants, whereas in the absence of NTFs, no significant difference in neuron survival between mutant and wildtype explants should be found. In the second hypothesis, the presence of trophic factors should lead to a similar survival rate of motoneurons in both mutant and wildtype explants, whereas in the absence of trophic factors, we should observe an increased susceptibility to endogenous death factors in *Necdin-*deficient explants. We therefore cultured ventral lumbar spinal cord explants from E12.5 mutant or wildtype embryos in the presence or in the absence of a cocktail of NTFs (Glial cell line Derived Neurotrophic Factor, GDNF; Brain-derived neurotrophic factor, BDNF and Ciliary Neurotrophic Factor, CNTF) and determined motoneuron survival by counting Islet-1/-2 positive cells (Figure 4A).

We found that in the presence of NTFs, the number of surviving motoneurons was not significantly different between wildtype and *Necdin-*deficient explants (*Necdin^+/+^*: 71 (59, 72), *n* = 5; *Necdin^+/−^*: 57 (56, 62), *n* = 5, n.s) (Figure 4B). However, in the absence of NTFs the number of surviving motoneurons appeared significantly different with a decrease of 43% of surviving cells in *Necdin-*deficient explants compared with wildtype explants (*Necdin^+/+^*: 26 (25, 29), *n* = 8; *Necdin^+/−^*: 17 (15, 21), *n* = 8; *P*<0.01) (Figure 4C). We therefore propose that Necdin acts by counteracting death signals rather than by potentiating the action of NTFs in motoneurons.

### TNFR1 expression correlates with Necdin expression in the lumbar spinal cord

**Figure 5:**
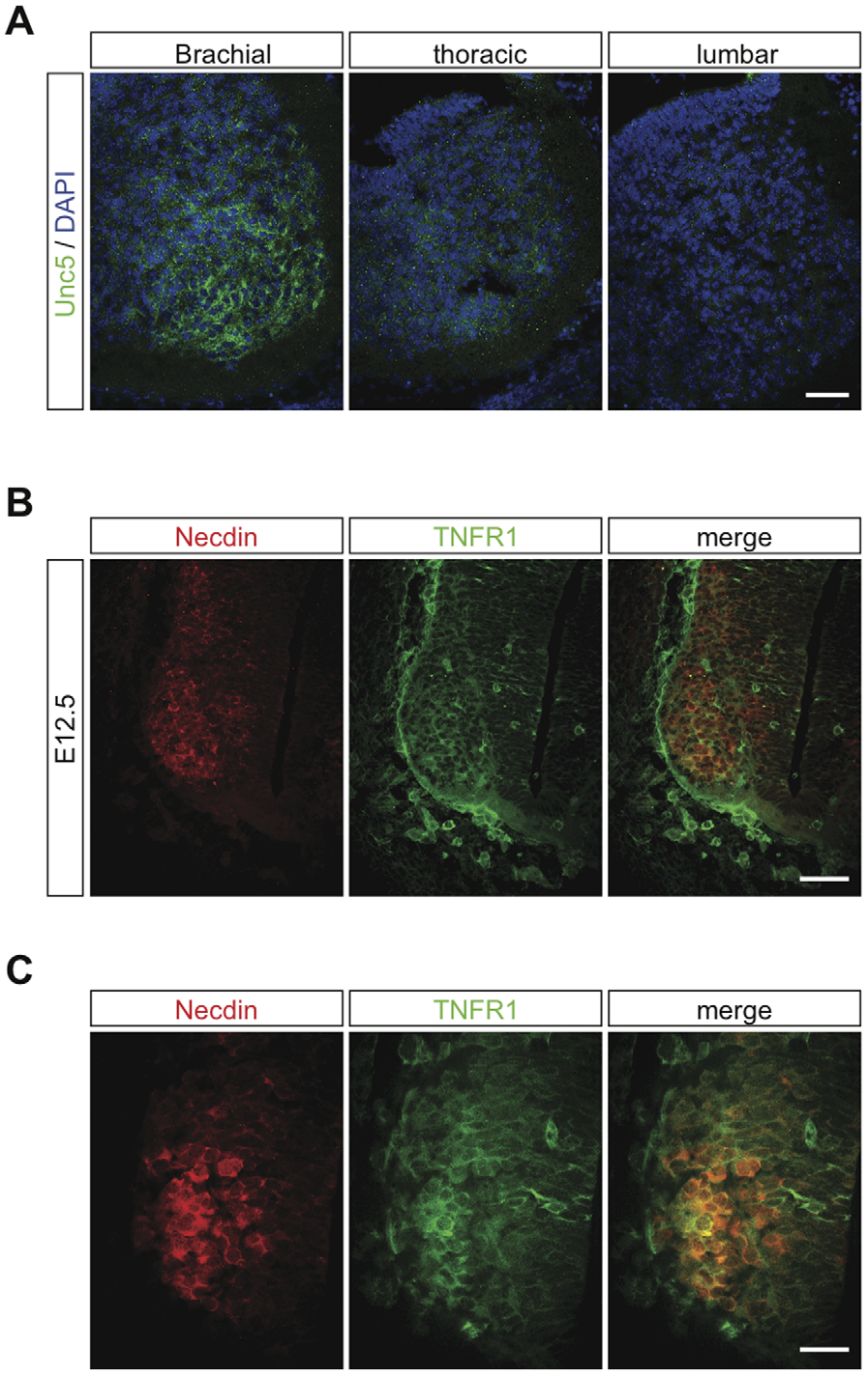
TNFR1 and Necdin are coexpressed in lumbar motoneurons at E12.5. (**A–C**) Immunohistochemistry analysis revealing the expression of the death receptors Unc5 (A) or TNFR1 (B and C), on different transversal sections corresponding to the brachial, thoracic and lumbar levels of E12.5 spinal cord. (A) Unc5 (green) is expressed in the brachial ventral horn of the spinal cord. Its expression is fainter in the thoracic ventral horn and is not detected in the lumbar ventral horn. (B) Necdin (red) and TNFR1 (green) are coexpressed in motoneurons of the lumbar region. (C) Higher magnification of (B) showing the colocalization of Necdin with TNFR1. Scale bar: A and B, 100 µm; C, 250 µm.

We next focused on the identity of the potential death receptor involved in the increased death of motoneurons in *Necdin*-deficient mice. A first potential candidate was the Unc5A receptor, since it has been shown to interact with MageD1, a MAGE gene with homology to Necdin, and to promote motoneuron death in the cervical spinal cord during PCD [32]. Since MageD1 is also expressed in lumbar motoneurons at E13.5 where, in contrast to Necdin, it plays a pro-apoptotic role [33], we hypothesized that Necdin might interact with Unc5A to interfere with the pro-apoptotic signal. Using an antibody specific to Unc5A, we analyzed its expression pattern, at E12.5, along the rostro-caudal axis. We detected a strong Unc5A immunoreactivity in the ventral part of the brachial spinal cord, which was reduced in the thoracic region. However, we were not able to detect any Unc5A expression in the lumbar region of the spinal cord (Figure 5A), indicating that Unc5A unlikely acts as a death receptor involved in the increase of PCD in *Necdin* mutants.

A second potential candidate was TNFR1. Indeed, it has been shown that, in the brachial region of the spinal cord, TNFα via TNFR1 commits developing motoneurons to cell death before PCD [34]. In addition, Necdin has been proposed to intervene in the TNFα pathway in myoblasts [35]. Interestingly, we found that TNFR1 was expressed in the ventral part of E12.5 spinal cords (Figure 5B) and showed that TNFR1-positive cells were Necdin-positive neurons (Figure 5C). Using mice expressing the green fluorescent protein (GFP) under the control of the motoneuron-selective Hb9 promoter (*Hb9::GFP*), we demonstrated that the TNFR1 positive cells were motoneurons (Figure S4A and Figure S5). It is noteworthy that we did not observe any TNFR1-positive cells in the lumbar region at E11.5, prior PCD (Figure S4B). This observation is consistent with a role of TNFR1 signaling in the death of lumbar motoneurons, which mainly take place between E12.5 and E13.5. Overall our observations suggest that Necdin might modulate the death promoting activity of TNFR1.

### Increase of motoneuron death in the absence of Necdin is linked to the TNFR1 pathway

**Figure 6:**
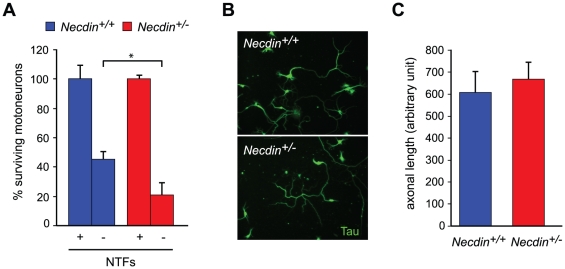
Necdin deficiency decreases motoneuron survival in absence of NTF but does not alter axonal growth. (A) Embryonic motoneurons isolated from *Necdin^+/+^*and *Necdin^+/−^* mice were cultured in the presence (+NTFs) or in the absence (−NTFs) of a cocktail of NTFs. After 24 hours, surviving motoneurons were counted and for each genotype survival is expressed as the percentage of the number of motoneurons surviving in the presence of NTF. In absence of NTFs a significant decrease of surviving motoneurons is observed in mutant compared to wildtype motoneurons. (B) When cultured for 3 days in the presence of NTFs Tau immunolabeling reveals a similar morphology between both genotypes. (C) When measuring, axonal length no difference is detected between mutant (*Necdin^+/−^*) and wildtype (*Necdin^+/+^*) motoneurons. Values are means ± S.D (in (A) or ± standard error of the mean in (B).

**Figure 7:**
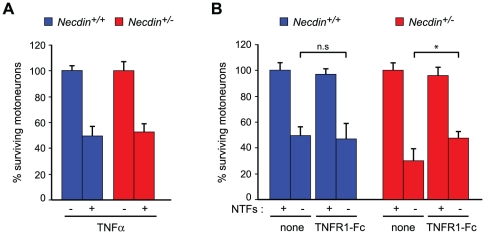
Increase of motoneuron cell death in absence of Necdin is linked to the TNFR1 pathway. (A) When cultured in presence of NTFs Necdin wildtype (*Necdin^+/+^*) and mutant (*Necdin^+/−^*) motoneurons are equally sensitive to death triggered by TNF alpha. Embryonic mutant and wildtype motoneurons were cultured in presence of NTFs for 24 hours and then treated or not with TNFα. After 48 hours of treatment motoneuron survival was assessed and expressed relative to non-treated cells. (B) In the absence of NTFs, TNFR1-Fc does not rescue cell death observed in wildtype motoneurons, but it restores the level of cell death in *Necdin* deficient motoneurons to the level observed in wild type cells. Wildtype or mutant motoneurons were cultured in presence (+) or absence (−) of NTFs and treated or not with 100 ng/ml of TNFR1-Fc 1 hours after seeding. Motoneuron survival was determined 24 hours after treatment and expressed as the percentage of the number of motoneurons surviving in the presence of NTF under same conditions. These results suggest that the increase of cell death observed in *Necdin*-deficient motoneurons is linked to the TNFα/TNFR1 pathway. Histograms show mean values ± S.D of triplicate wells in at least three independent experiments.

In order to investigate whether in the absence of Necdin TNFR1 signaling leads to an increase in motoneuron death, we used cultures of embryonic motoneurons isolated from E12.5 spinal cords. Consistent with the previous *in vivo* and *ex vivo* experiments, we found that in the absence of NTFs the percentage of surviving *Necdin*-deficient motoneurons was significantly lower than the percentage of surviving wildtype motoneurons cultured under the same conditions (*Necdin^+/+^*: 46.7 (44.4, 51), *n* = 3; *Necdin^+/−^*: 31.5 (21, 34.3), *n* = 3; *P*<0.05) (Figure 6A). We next measured the axonal growth in the presence of NTFs and found no difference between *Necdin*-deficient and wildtype motoneurons (Figure 6B,C). These data suggest that in the presence of neurotrophic support, *Necdin*-deficient and wildtype motoneurons are indistinguishable. Then, we checked the efficiency of TNFα at promoting cell death of mutant and wildtype motoneurons (Figure 7A). Motoneurons were cultured in presence of NTFs for 24 h and then treated for 48 hrs with TNFα at a concentration that has previously been demonstrated to efficiently kill motoneurons [36]. We observed that *Necdin-*deficient and wildtype motoneurons were killed at a similar rate (about 60%; data not shown). Since we proposed that Necdin acts by interfering with extrinsic death signals in the absence of NTFs, we performed a competition assay against endogenous TNFα in motoneuron cultures maintained in the presence or absence of NTFs, using a soluble chimeric protein (TNFR1-Fc) that impedes TNFα-TNFR1 interaction. In wildtype motoneurons, we observed that TNFR1-Fc did not influence motoneuron survival either in the presence or the absence of NTFs. In *Necdin*-deficient motoneurons, we observed that, in the absence of NTFs, TNFR1-Fc rescued motoneurons to the level observed in wildtype motoneurons, cultured under the same conditions (none: 31.5 (21, 34), *n* = 3 and TNFR1-Fc: 45.6 (41.3, 57.2), *n* = 3; *P*<0.05) (Figure 7B). By interfering with TNFα-TNFR1 signaling, we can restore a normal rate of death in mutant motoneurons, suggesting a role for Necdin in the TNFR1 signaling pathway. Altogether, our results provide evidence that Necdin contributes to motoneuron survival and suggest that Necdin tunes sensitivity to TNFR1 signaling in motoneurons.

## Discussion

This study aimed at investigating the contribution of Necdin in establishing and/or maintaining motor functions. Having previously observed that *Necdin*-deficient pups have a motor deficit that impairs hindlimb movement, we show here that Necdin plays an important role during the development of the motor system, *Necdin*-deficient mice lacking approximately 30% of their lumbar motoneurons.

**Figure 8:**
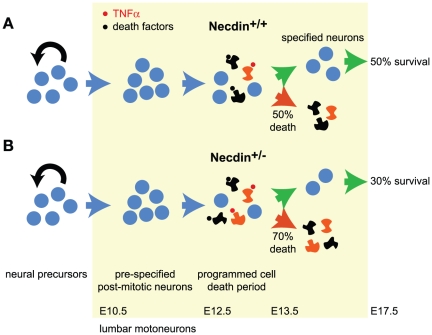
A proposed model for the anti-apoptotic role of Necdin in developing motoneurons. During embryogenesis, the development of motoneurons starts by a proliferation step in which Necdin is not expressed. These neuronal precursors differentiate into post-mitotic neurons and start to express Necdin (E10.5). These pre-specified motoneurons become sensitive to external death factors and in a normal situation (A) half of them will die and half of them will survive thanks to NTFs. In absence of Necdin (B), motoneurons are more sensitive to the TNFα death factor and an increase in cell death is observed.

Our first observation was that Necdin is expressed in all post-mitotic motoneurons along the rostro-caudal axis as early as E10.5 and that its expression is maintained until postnatal ages. At E17.5, which corresponds to the end of the period of naturally-occurring cell death in the lumbar spinal cords, we observed a loss of 31% of specified motoneurons in *Necdin*-deficient mice. However, at E11.5, prior to the period of PCD, the pools of post-mitotic motoneurons were similar between both mutant and wildtype mice. During the wave of PCD, we further revealed a significant 40% increase in cell death in the lumbar region of *Necdin*-deficient spinal cords (Figure 8). It is intriguing that although there is an expression of Necdin in all postmitotic spinal motoneurons, an increased developmental death is observed only at the lumbar level in *Necdin*-deficient mice. Indeed, at E12.5, quantification of the number of cleaved caspase-3 positive motoneurons in the brachial and thoracic spinal cord gave similar results between wildtype and mutant mice. Significantly, we previously reported an increase in PCD of developing sensory neurons specifically in the lumbar DRGs. Although, a concomitant sensory-motor pathology has already been described in spinal and bulbar muscular atrophy, distal spinal muscular atrophy type V or Charcot-Marie-Tooth disease type 2D [37], [38], restriction of the Necdin-associated pathology to the lumbar region remains unreported. Longitudinal gene profiling or proteomic analyses on different regions of the spinal cord during development would help us to identify factors governing such specificity.

### Necdin and the developmental cell death of motoneurons

Motoneurons are generated in excess in the developing spinal cord and about 50% of them are eliminated during a phase of programmed cell death (Figure 8A). Accumulating evidence suggests that motoneurons compete for access to limited quantities of neurotrophic support and that neurotrophic signaling in motoneurons prevents cell death cascades, including activation of caspases. Extrinsic death pathways, that mediate the activation of death signaling cascade, can be triggered by cell surface receptors such as Fas, TNFR, and p75^NTR^ following activation by their respective ligands. TNF α has been shown to play a role in developmental cell death [39], including brachial motoneuron cell death [34]. However during developmental PCD, the pattern of neuronal death in mice lacking TNFR1 is unchanged, suggesting that compensatory mechanisms might take place to complete the developmental program of motoneuron death [40]. Here we show that in the absence of *Necdin* there is an increase of motoneuronal death during the wave of PCD but no, or limited compensatory mechanisms take place.

Our results are in agreement with a model (Figure 8), in which newly generated post-mitotic motoneurons are committed to undergo programmed cell death very early, before they innervate their target muscles. The death program would not be completed if they gain access to trophic support (around 50% of them). In the remaining proportion of motoneurons that fail to access NTFs, the death program would be completed and cells eliminated. In the light of our results, we can propose that Necdin may act as an anti-apoptotic molecule that maintains post-mitotic motoneurons competent to respond to neurotrophic signals (Figure 8). Sedel, F. and colleagues proposed that TNFα can act as an extrinsic signal that commits motoneuron to a cell death fate [34], at least at the brachial level, two days before the period of cell death begins. Interestingly, at the brachial level, TNFR1 is transiently expressed during this critical period and we also showed a TNFR1 expression at E12.5 (and not at E11.5) in lumbar motoneurons. In the absence of Necdin, a TNF-dependent process could be exacerbated.

### Necdin and TNFα/TNFR1 pathway

Using cultures of E12.5 spinal cord explants and motoneurons, we observed that in the absence of NTFs, motoneuron survival was significantly lower (about 40%) in the absence of Necdin than in wildtype. This difference in neuron viability, which is similar to that observed *in vivo*, suggests that *Necdin*-deficient motoneurons have an increased susceptibility to death factor(s)/death receptors signaling. The first Necdin interactor to consider was the receptor p75^NTR^. p75^NTR^ is expressed in rat developing motoneurons during the wave of PCD [41], and also in the spinal cord of wild-type and *Necdin-*deficient embryos at E13.5 (data not shown). Necdin interacts with p75^NTR^ [15], [42] and a common signaling pathway involving both proteins and allowing the internalization of p75^NTR^ has been proposed [43]. However, mice lacking p75^NTR^ did not shown any loss of spinal cord motoneurons at P7 [44] neither an increase of PCD at E13.5 [33]. In developing sensory neurons of dorsal root ganglia of *Necdin*-deficient embryos, we previously described a similar increased cell death during the wave of PCD; however, this increased cell death does not affect the number of p75^NTR^ expressing neurons [27]. Altogether, these data indicate that Necdin might also function in a p75^NTR^-independent manner to prevent motoneuron developmental cell death *in vivo.*

Based on our data, we propose that the TNFα/TNFR death signaling pathway is involved in the increased cell death observed in *Necdin-*deficient motoneurons. It has previously been proposed that Necdin intervenes in the TNFα pathway [35]. The authors showed that in primary myoblasts isolated from *Necdin*-deficient mice, the TNFα pathway inhibits, at least in part, their myogenic differentiation. Interestingly, these mutant myoblasts expressed a higher amount of TNFR1 at the plasma membrane, as measured by fluorescence-activated cell sorting (FACS) analysis, although the quantity of the *Tnfr1* mRNA was not increased [35]. Taken together, these data suggest that an increase in TNFR1 receptors at the plasma membrane of motoneurons might also explain why *Necdin*-deficient motoneurons have an increased sensitivity to TNFα in the window of time in which PCD occurs. In our hands, primary cultured motoneurons did not survive FACS analysis and we were not able to reliably quantify the levels of TNFR1 receptor at the plasma membrane by immunolabeling.

### Necdin interaction with the TNFR1 signaling pathway

The constitutive or inducible TNFR1 release to the extracellular compartment plays a role in the control of TNFR1 at the cell membrane and can also regulate the TNFα activity by the generation of extracellular receptors that function as TNFα-binding proteins [45]. In human vascular endothelial cells, it has been shown that extracellular TNFR1 release requires the calcium-dependent formation of a Nucleobindin-2 (NucB2)-aminopeptidase regulator of TNFR1 shedding (Arts-1) complex associated with TNFR1 [46]. Interestingly, it has previously been shown that Necdin interacts with NucB2, thereby participating in the control of Ca^2+^ homeostasis in neuronal cytoplasm [18]. We therefore hypothesized that, indirectly, Necdin might control the TNFR1 release via regulation of the NucB2-Arts-1 complex.

First, we performed immunofluorescence confocal laser-scanning microscopy experiments in order to check whether NucB2 and Arts-1 are expressed in E12.5 developing motoneurons and to search for a colocalization of Necdin, NucB2, Arts-1, and TNFR1. We found that TNFR1 colocalized partially with Arts-1, NucB2 and Necdin in cultured E12.5 embryonic motoneurons (Figure S5 and Text S1, Supporting materials and methods). Because of the limited quantity of material obtained from primary culture of motoneurons, we were not able to perform immunoprecipitation assays with the different partners. In order to reveal in those motoneurons a functional role of the Arts-1-NucB2 complex in extracellular TNFR1 release, we then performed a proximity ligation assay (PLA) using the Duolink system to visualize potential physical interactions (10–30 nm) between TNFR1 and Arts-1, TNFR1 and NucB2 and TNFR1 and Necdin [47], [48] (Figure S5D–I). Necdin-NucB2 and NucB2-Arts1 pairs were not tested due to the overlapping species specificities of the available antibodies. Analysis of PLA signal was performed using confocal imaging and by counting the number of dots. We detected a high number of PLA signals for the TNFR1-Arts-1 pair, comparable with the positive control (Figure S5D,G,I). A lower number of dots was detected for the TNFR1-NucB2 pair (Figure S5E,I) and only few dots were counted for the TNFR1-Necdin pair (Figure S5F,I). These data suggest a physical interaction between TNFR1 and Arts1 and potentially TNFR1 and NucB2. These preliminary data suggest that the TNFR1/Arts-1/NucB2 complex might also exist in developing motoneurons, but we cannot demonstrate that Necdin interacts with NucB2 since appropriate antibodies for such an experiment are not available. In our hypothesis, Necdin would influence the release of TNFR1 into the extracellular space and/or the quantity of TNFR1 at the cell surface in motoneurons.

### Necdin and the motor deficit in Prader-Willi syndrome

PWS results from a loss of paternal expression of several contiguous imprinted genes located in the 15q11-q12 region. Recent studies support a major role of the *SNORD116* locus in the PWS phenotype, with three patients showing an overlapping microdeletion [49], [50], [51], encompassing the *SNORD116* genes. However, the question about the mechanism(s) by which these deletions, including the *SNORD116* locus, cause the physiopathology observed in these patients remains elusive. Necdin-deficient mice present defects reminiscent of specific PW symptoms and interestingly, at the cellular level, a same defect of polarization of the cytoskeleton is described in fibroblasts of PW patients and in mouse embryonic fibroblasts of *Necdin* deficient mice [25]. This observation reinforces the idea that the lack of Necdin is indeed responsible for some PW symptoms. It would be interesting to evaluate whether levels of Necdin in patient's tissues are altered by SNORD116.

The causes of the severe hypotonia, in early infancy, and of motor deficiency in later life, observed in PWS [52], are unresolved but relate to impairments in the nervous and muscular system and body composition. Our results reveal an important role of Necdin during motoneuron development, strongly suggesting that the lack of Necdin is involved in motor deficiency in PWS. The expression of NECDIN in human embryonic spinal cord is in accordance with this hypothesis [2]. Since the TNFα-TNFR1 pathway is involved in injury-induced apoptotic death of adult motoneurons, our study raises the question on the role of Necdin in motoneuron death following nerve injury or in neurodegenerative diseases. Interestingly, in a transgenic mouse model of amyotrophic lateral sclerosis (ALS) expressing the G93A mutation of superoxide dismutase-1, a modulation of Necdin expression has been documented. Necdin was found to be upregulated at the presymptomatic stage and downregulated at the end stage, suggesting that Necdin might contribute to the neurodegenerative process in ALS [53].

## Materials and Methods

### Ethics statement

All breeding and experiments were carried out in accordance with the European and National guidelines for the care and use of laboratory animals (Council Directive 86/6009/EEC).

### Breeding and genotyping of *Necdin*-deficient mice

Necdin-deficient mice were generated as previously described [6]. We used a mouse colony generated on the C57BL/6J background (more than 10 backcrosses). Because Necdin is an imprinted gene, only paternally expressed, we crossed heterozygote males (*Necdin* ^−m/+p^) with wildtype C57BL/6J females, in the generated litters, half the embryos were control (*Necdin* ^+/+^) and half were functionally *Necdin-*deficient (*Necdin^+/−^*). The age of the embryos was determined at 9:00 am by the presence of a vaginal plug in the pregnant mothers and considered as embryonic day 0.5 (E0.5). For motoneuron cultures, embryos were kept in Hibernate E (Invitrogen, Carlsbad, CA, USA) at +4°C during their genotyping. All embryos were genotyped by PCR with the following primer sets: Nec F, 5′-TCTCATGCTTGAACTGCA-3′ and Nec B, 5′- CAGGTAATTCTGCTGGAC-3′; a 1503 bp and a 228 bp fragment were generated, which correspond to the wildtype or mutant allele respectively. PCR conditions were: 94°C 1 mn then 35 cycles: 94°C 20 sec, 56°C 20 sec, 72°C 45 sec.

### Immunohistochemistry

Embryos or explants were collected, fixed and sectioned using a cryostat (10 or 12 µm thick) as previously described [27]. Sections were blocked for 1 hour at room temperature in phosphate buffered saline (PBS) containing 10% heat-inactivated goat or donkey serum, 0.5% Triton X-100 and incubated overnight at +4°C with primary antibodies diluted in PBS containing 10% heat-inactivated goat serum and 0.1% Triton X-100. The primary antibodies were: rabbit polyclonal anti-Necdin (07-565; Millipore, Bedford, MA, USA; 1∶500), mouse monoclonal anti-Islet-1 and Islet-2 (2D6 and 4D5; Developmental studies Hybridoma Bank of Iowa University; 1∶500 and 1∶100 respectively), rabbit polyclonal anti-cleaved-caspase-3 (9661, Cell Signaling Technology, Beverly, MA, USA; 1∶500), rabbit polyclonal anti-Unc5 (ab39654, Abcam, 1∶500), mouse monoclonal anti-TNFR1 (sc-8436, Santa Cruz Biotechnology, Santa Cruz, CA, USA, 1∶500). Sections were washed 3 times in PBS and incubated for 1 hr at room temperature with secondary antibodies diluted in 5% Goat serum, 1% BSA, 0.3% Triton X-100 in PBS. Biotinylated secondary antibodies and the ABC complex from the Vectastain kit (Vector Laboratories, Burlingame, CA, USA), were used for detection. Alternatively, fluorochrome-conjugated secondary antibodies were used (Jackson Immunoresearch laboratories, West Grove, PA, USA). Sections were examined on a Zeiss Axioplan 2 microscope with an Apotome module.

### TUNEL assay

To identify cells undergoing apoptosis, the terminal deoxynucleotidyl transferase-mediated biotinylated dUTP nick end labeling (TUNEL) technique was used. Whole-mount TUNEL was performed essentially as previously described [30]. Spinal cords were fixed, dehydrated, and rehydrated through graded ethanol concentrations into PBS and then stained with the ApopTag kit (Oncor Gaithersburg, MD, USA). Spinal cords were incubated in ApopTag equilibration buffer for 5 min at room temperature and transferred to the working strength TdT enzyme solution for 12 hr at 4°C, followed by 2 hr at 37°C. The reaction was stopped by incubating the spinal cords in ApopTag stop solution for 40 min at 37°C. After washing in TBST (0.14 M NaCl, 10 mM KCl, 25 mM Tris, pH 7.0, and 0.1% Tween-20), endogenous AP was inactivated by incubating the spinal cords in TBST for 20 min at 65°C. The spinal cords were then incubated in blocking solution (10% goat serum, 1% BSA in PBS) followed by an overnight incubation at 30°C with an anti-DIG-AP conjugate (Roche Diagnostics, Indianapolis, IN, USA; diluted 1∶2000 in blocking solution). They were then extensively washed in MABT, stained as described for the whole-mount in situ hybridization [27], and examined under transillumination. Several spinal cords from wildtype and mutant embryos, issued from the same litter, were examined. TUNEL positive cells were counted in the lumbar region specifically as previously described [30].

### Spinal cord explants

Lumbar spinal cord regions were dissected from E12.5 (somites counted) wildtype or Necdin-deficient embryos issued from the same litter. We excluded the ventral part of the dorsal root ganglia with the corresponding part of the sclerotome. From each lumbar region dissected, two sections were made to create three explants. The dissection and culture of explants was performed as previously described [34]. Briefly, these explants were grown in four-well culture dishes on coverslips previously coated with polylysin/laminin in Neurobasal medium containing 20% FBS and NGF (100 g/ml). Culture medium [34] was complemented with (+NTF) or without (−NTF) a cocktail of NTFs (GDNF, 1 ng/ml; CNTF, 10 ng/ml and BDNF, 10 ng/ml). After two days *in vitro* (DIV), explants were fixed for 10 min at RT in 4% paraformaldehyde in PBS, washed 3 times in cold PBS, and incubated overnight in 20% sucrose at +4°C. The explants were then frozen in a Tissue-Teck cryostat (Oxford laboratories) and stored at −80°C. Cryosections (12 µm) were performed for each animal (3 explants/lumbar spinal cord) and immunolabeled with anti-Islet-1/-2 antibodies as indicated above. An average of 25 sections was obtained for each explant. The number of Islet-1/-2 positive cells was counted for each section and the mean of these values (25 sections×3 explants) was calculated for each lumbar spinal cord. Values in Figure 4B represent the mean values of sections per animal.

### Cell survival

For motoneuron quantification *in vivo*, spinal cord sections (10 µm each, collected every fourth sections) were immunostained with the motoneuron marker Islet-1/-2 as indicated above. Only the cells located in the ventral part were considered as motoneurons. The interneurons located in the medio-dorsal part were separately counted at E17.5.

For caspase-3 quantification in the lumbar region *in vivo*, the number of cleaved caspase-3 positive cells was counted on each transversal section (10 µm), and the mean number of caspase-3 positive cells was determined per section. For the quantification of cleaved caspase-3, counting was done directly on whole mount spinal cords immunolabeled with anti-cleaved-caspase-3 antibodies.

For motoneuron culture, E12.5 *Necdin^+/+^* and *Necdin^+/−^* embryos were collected from the same mother and genotyped by PCR as described above prior to dissection of the spinal cord. For *Hb9::GFP* motoneuron cultures, E12.5 transgenic embryos were sorted under a fluorescence microscope prior to dissection of the spinal cord. Embryonic motoneurons were isolated as described [54], [55], using iodixanol density gradient centrifugation. Motoneurons were plated on poly-ornithine/laminin-treated wells in supplemented Neurobasal medium at a density of 1,100 cells/cm^2^ for survival assays and 2,600 cells/cm^2^ for immunofluorescent labeling. Neurons were cultured in the presence (or not, when indicated) of a cocktail of NTFs (GDNF, 0.1 ng/ml; BDNF, 1 ng/ml and CNTF 10 ng/ml) [54], [55]. When indicated motoneurons were treated at indicated times with 100 ng/ml of TNFR1-Fc (Alexis Biochemicals, San Diego, CA, USA) or 100 ng/ml of TNFα (BD Biosciences, Franklin Lakes, NJ, USA) and survival was determined at indicated times under light microscopy using morphological criteria [54]. To compare values from different experiments in a quantitative manner, the number of motoneurons cultured in the presence of neurotrophic factors was taken as 100% survival.

### Neurite outgrowth analysis

Neurons were fixed three days post seeding with 4% paraformaldehyde in PBS and immunostained with rabbit polyclonal anti-Tau antibodies (ab64193, Abcam, Cambridge, MA, USA, 1∶200) labeling being revealed using a Cyanine 3 anti-rabbit antibody (Jackson Immunoresearch, 1∶500) and a mouse monoclonal anti-GFP (NB600-597, Interchim, 1∶500) antibody revealed using an Alexa anti-mouse antibody (1/1000). Based on the immuno labeling and using Metamorph software, the total length of neurites was measured and compared between mutant and wildtype motoneurons.

### Western blotting

Lumbar ventral spinal cords were dissected at different embryonic and postnatal developmental stages from wildtype and *Necdin* mutant mice. Tissues were snap frozen in liquid nitrogen and stored at −80°C. Protein extractions were performed as indicated previously [56]. Homogenate samples containing equal amount of proteins (30 µg as determined using BCA Protein Assay Reagent, Thermo scientific, Waltham, MA,USA) were boiled for 5 min in SDS buffer (50 mM Tris-HCl, pH 6.6, 2% SDS, 10% glycerol, 0.1% bromophenolblue, and 5% β-mercaptoethanol), and separated on a 10–20% SDS/polyacrylamide gel (Biorad) in Tris-Tricine buffer. After migration, proteins were transferred onto 0.2 µm nitrocellulose membranes (Millipore). Membranes were blocked for 1 hour at RT with TBS-T buffer (150 mM NaCl, 20 mM Tris HCl, pH 7.4, 0.1% Tween-20) containing 5% milk. The membranes were then incubated overnight with polyclonal antibodies against Necdin diluted at 1∶500 in TBS-T containing 2.5% milk. Membranes were washed three times in TBS-T and incubated for 1 hour with an anti-rabbit horseradish peroxydase antibody. The proteins were visualized by enhanced chemiluminescence (ECL plus, GE Healthcare, Buckinghamshire, UK). Each blot was re-probed using an anti mouse anti-Actin (Sigma-Aldrich, St Louis, MO, USA) antibody, as loading control.

### Statistical analyses

According to the sample size, we used non-parametric statistical analysis. Statistical comparisons between independent samples were made using the Wilcoxon-Mann-Whitney rank sum test (StatXact software, Cytel Inc., Cambridge, MA, USA). Values are indicated as following: (Q2 (Q1, Q3), *n*, *P* value), where Q2 is the median, Q1 is the first quartile and Q3 is the second quartile. The level of significance was set at a *P* value less than 0.05.

## Supporting Information

Figure S1 **The absence of** ***Necdin*** **leads to motor deficit in mice.** (A) At P8, a difference in the body size and in position of the hindpaws is observed between wildtype (*Necdin^+/+^*) and *Necdin*-deficient (*Necdin^+/−^*) pups. The *Necdin^+/−^* pups are unable to flex and move their hindlimbs. (B) In adulthood, the *Necdin*-deficient (*Necdin^+/−^*) mice have a reduced performance in the rotarod test in comparison with the wildtype (*Necdin^+/+^*) mice.
(EPS)

Figure S2 ***Necdin*** **mRNA is expressed in the spinal cord during development.** (A) Immunostaining of brachial and lumbar spinal cord of *Hb9::GFP* E12.5 embryos using antibodies against Necdin (in red). (B) *In situ* hybridization analysis was conducted, as previously described (Andrieu et al., 2006), to detect *Necdin* mRNA in the spinal cord at indicated developmental stages. (C) Combined immunohistochemical labeling (Islet-1/-2) and in situ hybridization (*Necdin*) on E12.5 spinal cord sections. Scale bar in (A), (B), 100 µm and (C), 20 µm.
(TIF)

Figure S3 **The number of Islet-1/-2 positive cells in the medio-dorsal part of the spinal cord is not modified in the** ***Necdin*** **-deficient embryos.** (A) At E17.5 anti-Islet-1/-2 antibody recognizes ventral motoneurons and a pool of interneurons located in the medio-dorsal part of the spinal cord. (B) A quantification of these Islet-1/-2 positive interneurons shows no difference between both genotypes throughout the rostro-caudal level.
(PDF)

Figure S4 **TNFR1 is expressed in the lumbar region of the spinal cord at E12.5 but not at E11.5.** Immunohistochemistry analysis revealing the expression of TNFR1 (in red) (A and B) and Hb9 (*Hb9::GFP*) (in green), on different transversal sections corresponding to lumbar levels of spinal cord at E12.5 (A) and E11.5 (B). TNFR1 and Hb9 are coexpressed in ventral motoneurons at E12.5 (A) but not at E11.5 (B). Transverse sections were countered stained with DAPI (in blue).
(TIFF)

Figure S5 **Detection of interactions between TNFR1 and the Arts1-NucB2 complex and Necdin.** (A–C) Immunofluorescence labeling of endogenous TNFR1 (in red), Arts1, NucB2 and Necdin (in green) in primary cultures of embryonic motoneurons. Motoneurons are visualized by the expression of GFP (in blue) under the control of the *Hb9* promoter. (D–H) PLA labeling pattern of TNFR1-ARTS1 pair (D), TNFR1-Nuc B2 pair (E), TNFR1-Necdin (F) pair in embryonic motoneurons (in green, *Hb9::GFP*). (G) As a positive control, an anti-TNFR1 primary antibody was used in combination with anti-mouse PLA PLUS and anti-mouse PLA MINUS probes. (H) As a negative control, an anti-TNFR1 primary antibody was used in combination with anti-mouse PLA PLUS and anti-rabbit PLA MINUS probes. (I) Box-and-whisker plot showing the number of PLA dots for each indicated pair (I). Scale bar, 20 µm.
(TIFF)

Text S1 **Supporting materials and methods.**
(DOC)
