# Supplementary figures and images for: Necdin Protects Embryonic Motoneurons from Programmed Cell Death
Source: PLoS One. 2011 Sep 2;6(9):e23764. doi: 10.1371/journal.pone.0023764 (PMC3166279; doi:10.1371/journal.pone.0023764)

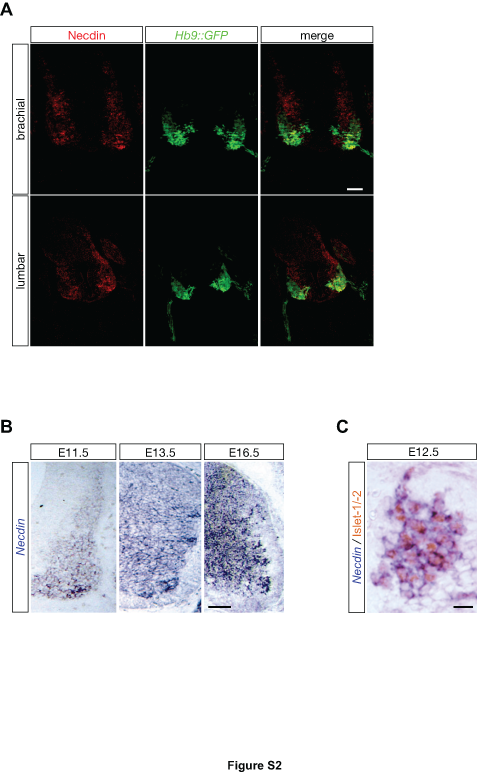

Supplement: Figure S2 — Necdin mRNA is expressed in the spinal cord during development. (A) Immunostaining of brachial and lumbar spinal cord of Hb9::GFP E12.5 embryos using antibodies against Necdin (in red). (B) In situ hybridization analysis was conducted, as previously described (Andrieu et al., 2006), to detect Necdin mRNA in the spinal cord at indicated developmental stages. (C) Combined immunohistochemical labeling (Islet-1/-2) and in situ hybridization (Necdin) on E12.5 spinal cord sections. Scale bar in (A), (B), 100 µm and (C), 20 µm. (TIF) [file pone.0023764.s002.tif]

**A**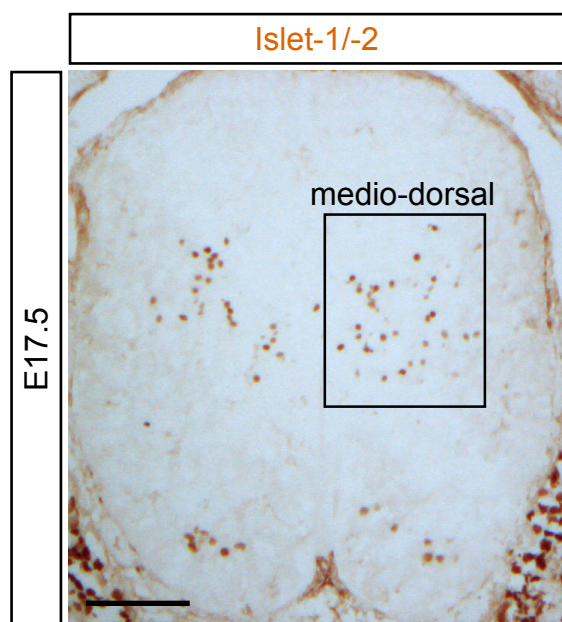**B**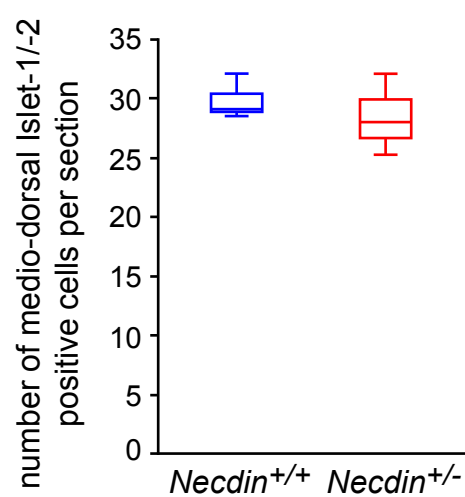

Supplement: Figure S3 — The number of Islet-1/-2 positive cells in the medio-dorsal part of the spinal cord is not modified in the Necdin -deficient embryos. (A) At E17.5 anti-Islet-1/-2 antibody recognizes ventral motoneurons and a pool of interneurons located in the medio-dorsal part of the spinal cord. (B) A quantification of these Islet-1/-2 positive interneurons shows no difference between both genotypes throughout the rostro-caudal level. (PDF) [file pone.0023764.s003.pdf]

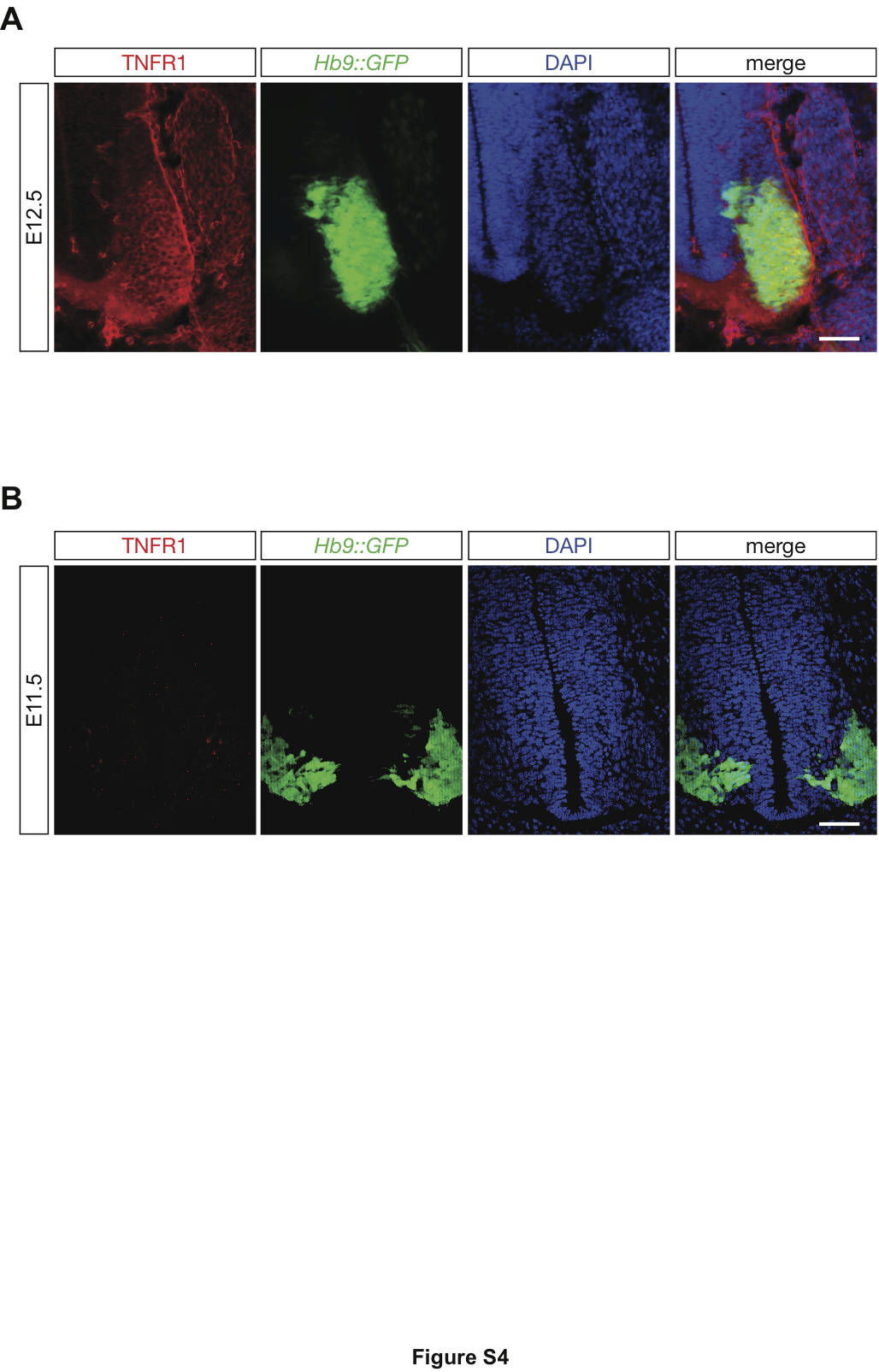

Supplement: Figure S4 — TNFR1 is expressed in the lumbar region of the spinal cord at E12.5 but not at E11.5. Immunohistochemistry analysis revealing the expression of TNFR1 (in red) (A and B) and Hb9 (Hb9::GFP) (in green), on different transversal sections corresponding to lumbar levels of spinal cord at E12.5 (A) and E11.5 (B). TNFR1 and Hb9 are coexpressed in ventral motoneurons at E12.5 (A) but not at E11.5 (B). Transverse sections were countered stained with DAPI (in blue). (TIFF) [file pone.0023764.s004.tiff]

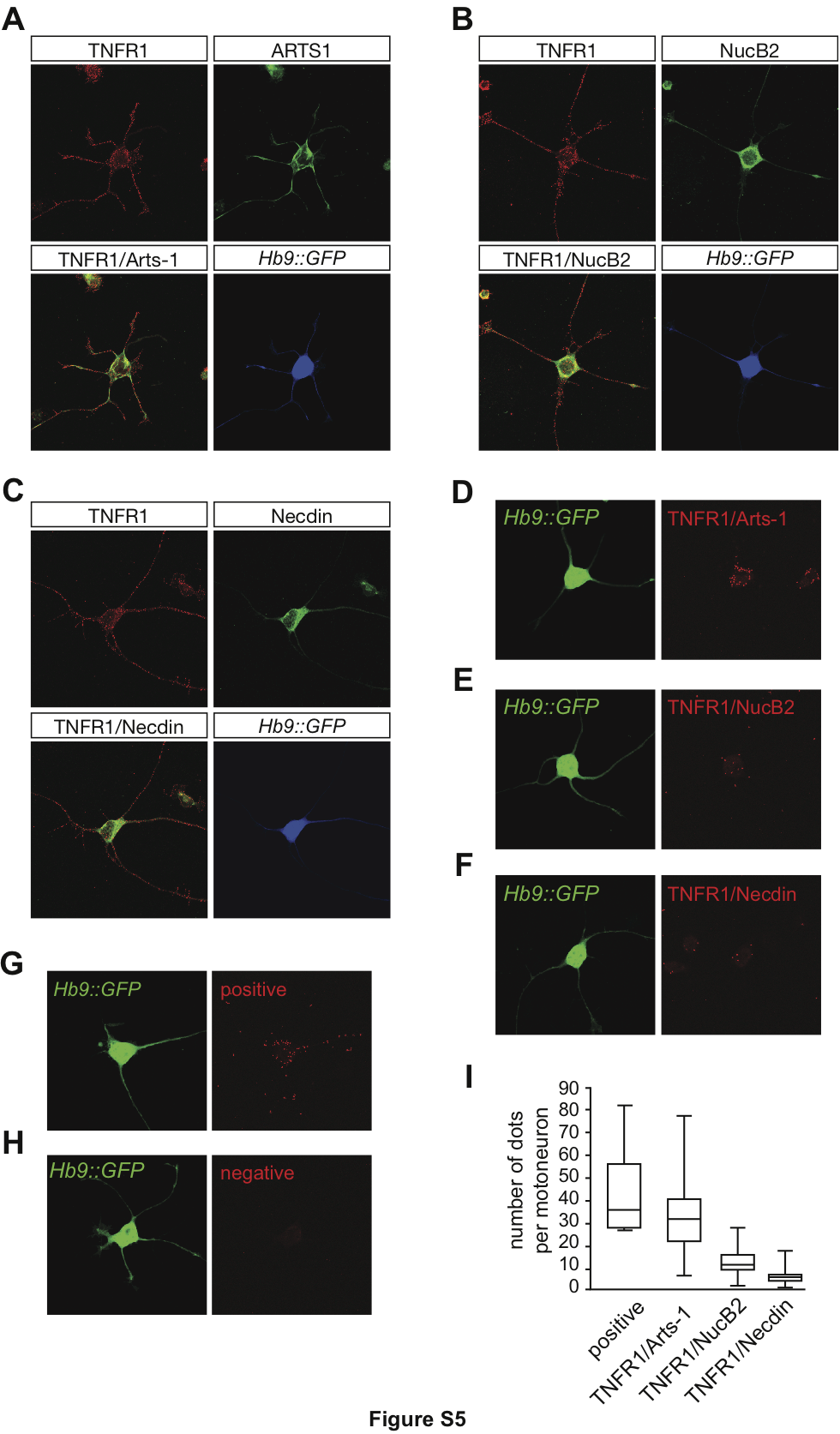

Supplement: Figure S5 — Detection of interactions between TNFR1 and the Arts1-NucB2 complex and Necdin. (A–C) Immunofluorescence labeling of endogenous TNFR1 (in red), Arts1, NucB2 and Necdin (in green) in primary cultures of embryonic motoneurons. Motoneurons are visualized by the expression of GFP (in blue) under the control of the Hb9 promoter. (D–H) PLA labeling pattern of TNFR1-ARTS1 pair (D), TNFR1-Nuc B2 pair (E), TNFR1-Necdin (F) pair in embryonic motoneurons (in green, Hb9::GFP). (G) As a positive control, an anti-TNFR1 primary antibody was used in combination with anti-mouse PLA PLUS and anti-mouse PLA MINUS probes. (H) As a negative control, an anti-TNFR1 primary antibody was used in combination with anti-mouse PLA PLUS and anti-rabbit PLA MINUS probes. (I) Box-and-whisker plot showing the number of PLA dots for each indicated pair (I). Scale bar, 20 µm. (TIFF) [file pone.0023764.s005.tiff]
