# Supplementary material for: Necdin Protects Embryonic Motoneurons from Programmed Cell Death
Source: PLoS One. 2011 Sep 2;6(9):e23764. doi: 10.1371/journal.pone.0023764 (PMC3166279; doi:10.1371/journal.pone.0023764)
Supplement: Supplementary file 2 [file pone.0023764.s006.doc]

Text S1

Rotarod test
Mice of indicated genotype were first given three habituation trials for 60 sec each (one trial at 0 rpm and 2 trials at 4 rpm). Animals were then submitted to 4 testing trials separated by 5-10 min interval and during which the rotation speed accelerated from 4 to 40 rpm in 5 min.

Immunocytochemistry and In situ Proximity Ligation Assay
Hb9::GFP motoneurons were cultured in the absence of NTFs for 6 hours on glass coverslips, fixed in 4% Antigenfix (Diapath, Martinengo, Italy) for 20 minutes at +4°C. Cells were incubated for one hour at RT in PBS containing 4% bovine serum albumin, 4% horse serum and 0.1% Triton X-100 in PBS. Cells were coimmunolabeled over night in blocking solution at +4°C with primary mouse monoclonal antibody to TNFR1 (1:500) in combination with either rabbit polyclonal antibody to Arts-1 (a gift from Dr. Stewart J. Levine, NIH, Bethesda, USA ; 1:1000), rabbit polyclonal antibody to NucB2 (H-003-22 ; Phoenix Pharmaceuticals, Burlingame, CA, USA ; 1:500) or rabbit polyclonal antibody to Necdin (1:500). Alexa Fluor 555-conjugated donkey anti-mouse and Alexa Fluor 647-conjugated donkey anti-rabbit were used as secondary antibodies (Invitrogen). Z-Stack images were taken using a Zeiss LSM510 laser scanning confocal microscope.
To detect the interaction between TNFR1 and Arts-1, NucB2 or Necdin, the DuoLinkTM in situ Proximity Ligation Assay (PLA) (Eurogentec, Seraing, Belgium) was used according to the manufacturer’s instructions. Briefly, Hb9::GFP motoneurons were cultured, fixed, and incubated with primary antibodies as mentioned above. The secondary antibodies with attached PLA probes (anti-mouse PLA PLUS, anti-Mouse PLA MINUS and anti-Rabbit PLA MINUS) were supplied in the Duolink II kit and diluted as indicated by the manufacturers in primary antibody incubation buffer. Duolink II Detection Reagents Red was used for amplification and glass coverslips were then mounted using Vectashield mounting media for Fluorescence (Vector Labs). Z-Stack images were taken using a Zeiss LSM510 laser scanning confocal microscope or a Zeiss Axioplan 2 microscope with an Apotome module and fluorescence signals were counted using Axiovision software. A fluorescence signal indicates that two proteins within cells are separated by < 40 nm (www.olink.com).
